# The Effects of Exercise on Cognitive Recovery after Acquired Brain Injury in Animal Models: A Systematic Review

**DOI:** 10.1155/2015/830871

**Published:** 2015-10-05

**Authors:** Elise Wogensen, Hana Malá, Jesper Mogensen

**Affiliations:** The Unit for Cognitive Neuroscience, Department of Psychology, University of Copenhagen, Oester Farimagsgade 2A, 1354 Copenhagen K, Denmark

## Abstract

The objective of the present paper is to review the current status of exercise as a tool to promote cognitive rehabilitation after acquired brain injury (ABI) in animal model-based research. Searches were conducted on the *PubMed, Scopus*, and *psycINFO* databases in February 2014. Search strings used were: exercise (and) animal model (or) rodent (or) rat (and) traumatic brain injury (or) cerebral ischemia (or) brain irradiation. Studies were selected if they were (1) in English, (2) used adult animals subjected to acquired brain injury, (3) used exercise as an intervention tool after inflicted injury, (4) used exercise paradigms demanding movement of all extremities, (5) had exercise intervention effects that could be distinguished from other potential intervention effects, and (6) contained at least one measure of cognitive and/or emotional function. Out of 2308 hits, 22 publications fulfilled the criteria. The studies were examined relative to cognitive effects associated with three themes: exercise type (forced or voluntary), timing of exercise (early or late), and dose-related factors (intensity, duration, etc.). The studies indicate that exercise in many cases can promote cognitive recovery after brain injury. However, the optimal parameters to ensure cognitive rehabilitation efficacy still elude us, due to considerable methodological variations between studies.

## 1. Introduction

Physical exercise has long been known to be effective in the treatment and prevention of many physical conditions such as type 2 diabetes, hypertension, obesity, dyslipidemia, and cardiovascular disease [1–3]. Furthermore, exercise has been found to reduce symptoms of depression and anxiety [4–7]. Exercise has also garnered considerable interest as a tool to promote cognitive health. Studies of healthy older adults have shown positive effects of exercise on measures of cognitive function [8, 9]. Research into the effects of physical activity on enhancing cognitive/academic abilities in children shows some promise. However, the findings are still fairly limited and more randomized, controlled trials are needed [10–12]. Similarly, there is some evidence that physical activity can improve cognition or prevent mental decline in people with neurological and neurodegenerative disorders. The overall results, however, remain inconclusive due to differences in methodologies and quality of studies [13–16].

Physical exercise after acquired brain injury (ABI) has received attention as a cost-effective, noninvasive, and practicable rehabilitation tool. Preclinical research has shown that post-ABI exercise can increase cerebral growth factor levels [17–21], reduce apoptosis-related processes [22–24], promote neurogenesis, neuronal survival, and regeneration [25–28], reduce lesion size [29, 30], modulate inflammatory responses [31], reduce astrocytosis [32, 33], and improve cerebral blood flow [34, 35]. However, less is known about the potential effects of exercise on cognitive recovery after ABI. Cognitive dysfunctions after brain injury, such as memory, attentional, and executive function impairments, are common and can negatively affect work performance, social competencies, and experienced quality of life [36].

In this paper, the preclinical research investigating the effects of post-ABI exercise on cognitive recovery will be systematically reviewed. Within brain injury rehabilitation, several factors (e.g., timing, repetition, intensity) have been shown to be of importance for promoting brain plasticity mechanisms and enhancing recovery outcome [37]. Such factors are also believed to be essential when using exercise as a cognitive rehabilitation tool. In the following, parameters that are believed to play a role in the efficacy of exercise, including type of exercise, starting point, and dose-related issues, will be examined.

## 2. Inclusion Criteria

Relevant research studies were found using the search terms “exercise (and) animal model (or) rodent (or) rat (and) traumatic brain injury (or) cerebral ischemia (or) brain irradiation,” all in all 9 search strings. The searches were performed in February of 2014 on the* PubMed*,* Scopus*, and* PsycINFO* databases, providing a total of 2308 hits. Articles were then selected using the following inclusion criteria:

- In English.
- Animal model based.
- Employing adult animals (rat models: min. 7 weeks old or min. 200 g; mouse models: min. 6 weeks old or min. 20 g; gerbil models: min. 11 weeks old or min. 55 g).
- Animals were subjected to acquired brain injury (ABI) in their adult life, either through mechanical injury, neurotoxic injection, irradiation, or induction of cerebral ischemia.
- Exercise was used as an intervention/treatment tool* after* cerebral injury (habituation to the exercise apparatuses prior to injury was accepted).
- The exercise regimens consisted of a general motor activation of all of the animals' extremities (i.e., running, swimming). Sole training of a single muscle group or extremity (i.e., forced limb use, grip training) was not included.
- The effects of the exercise intervention could be clearly distinguished from effects of nonexercise interventions if such were also investigated.
- Studies contained at least one measure of cognitive and/or emotional function after (or during) exercise treatment. Studies solely investigating motor abilities (i.e., balance tests, physical strength tests) or neural/molecular mechanisms were excluded.

Twenty-two research articles fulfilled the above inclusion criteria. Examination of the references in these articles did not uncover further publications that fulfilled the inclusion criteria.

Of the 22 papers, 14 used rats, five used mice, and three used gerbils as their experimental subjects. All used male animals except two (see Table 1). Regarding type of brain injury, eight were ischemia models (common carotid artery occlusion, middle cerebral artery occlusion, and photothrombosis), five used cortical impact injury, four used fluid percussion injury, one used closed head injury equipment, another used neurotoxic injection, and three used gamma irradiation.

Experimental groups fell into four types of exercise: nonmotorized running wheel exercise (nine studies), motorized treadmill exercise (11 studies), motorized running wheel (one study), swimming in a circular pool (one study), and swimming or running wheel exercise (one study).

Cognitive measures applied in these studies were spatial learning/retention paradigms administered in a water maze (12 studies) or in a Barnes maze (one study), visual discrimination and retention in a water maze (one study), object recognition tests (three studies), an object location test (one study), conditioning based learning paradigms (seven studies) (i.e., contextual fear learning, step-down avoidance task, passive avoidance task, stop-signal reaction time task, conditioned learning in a Y-maze), open field tests (three studies), and tail suspension tests (two studies). Some studies used more than one test.

## 3. Voluntary or Forced?

Within animal model based research, exercise is often differentiated into voluntary or forced paradigms. In voluntary paradigms, the animals are given a choice between movement and inactivity while having access to the exercise apparatus. In forced exercise, activity levels are controlled by external factors. Exercise in a nonmotorized running wheel allows animals to exercise at their own accord, while motorized treadmill running/running wheel exercise and swimming exercise do not offer such movement autonomy. The following section examines whether the type of exercise (voluntary or forced) exerts differential effects on cognitive recovery after ABI.

### 3.1. Voluntary Exercise

Nine studies included experimental groups subjected to voluntary (running wheel) exercise.

Wu et al. [38] subjected rats to lateral fluid percussion injury (lFPI) immediately followed by 12 days of running wheel exercise (7 of those days prior to cognitive testing), a diet high in docosahexaenoic acid (DHA), or both. They found that lFPI exercised animals did significantly better in a spatial learning task in a water maze (as shown by reduced latency to find a platform) in comparison to nonexercised lFPI animals kept on a normal diet. The DHA diet was also associated with improved spatial learning. Furthermore, injured rats on the combined exercise and DHA diet significantly outperformed all other lFPI groups. Molecular analysis showed increased levels of DHA, Acox1, and 17*β*-HSD4 (enzymes involved in DHA metabolism), Sir2 (involved in mitochondrial function), iPLA2 (molecules involved in membrane homeostasis), p-TrkB (BDNF receptor), and lower levels of 4-HHE (marker for lipid peroxidation) in the groups subjected to either exercise or the DHA diet (compared to controls) and a further increase/decrease in the combined group. The combined group also showed increased STX-3 (also involved in membrane homeostasis) and brain derived neurotrophic factor (BDNF) levels. The study indicates that early initiated voluntary exercise and/or the DHA diet can positively affect cognitive recovery after TBI, possibly through counteracting membrane damage and coordinating DHA metabolism.

Contrary to these results, Griesbach et al. [39] found that animals exposed to lFPI and early initiated exercise (from post-injury day 0) performed significantly worse on a spatial acquisition task in a water maze than all other groups, including an lFPI group starting exercise at a later point in time (at postinjury day 14) and lFPI nonexercised controls. Animals exercised later performed at the level of the sham operated animals. During a retention test (probe trial), all lFPI animals performed worse than noninjured animals, regardless of exercise treatment. The late exercise and sham groups showed increased hippocampal levels of the transcriptional regulator phosphorylated cyclic AMP response element-binding protein (pCREB) and BDNF. Moreover, there was a positive correlation between BDNF-levels and the amount of exercise. No BDNF-increase was seen in the early exercised group, which also showed lower levels of Synapsin-I (involved in synaptic vesicle clustering and release) and CREB.

Also finding detrimental effects, Crane et al. [40] subjected animals to a cortical contusion injury immediately followed by a 7-day running wheel exercise regimen. In a complex stop-signal reaction time task (a conditioning-based learning task requiring either inhibition or execution of a learned behavior depending on stimuli given), the exercised animals performed significantly worse for the first five test days compared with both the nonexercised injured animals and the sham animal groups. However, after a week of testing, the exercised animals returned to their baseline levels. The contused, exercised animals showed larger inflammatory responses (more GFAB and IBA1 positive cells) in the cortex and hippocampus, respectively. All contused groups had fewer surviving cells (less DAP1 positive cells) in the cortex, hippocampus, mediodorsal nucleus of the thalamus, and corpus callosum compared to the nonexercised, sham animals.

The conflicting results of the two first studies are somewhat surprising, as they use similar models and setups. Discrete differences, for example, in the duration of the exercise protocol, could potentially account for these divergent findings. While the studies by Griesbach et al. [39] and Crane et al. [40] both found detrimental effects of early exercise on cognitive performance, it appears that these effects are transient, as the animals seem to catch up during the rather short-time course of task acquisition.

Initiating exercise a little later after injury, Luo et al. [41] subjected C57/BL6 mice to middle cerebral artery occlusion (MCAO) followed by a one week postinjury break. Subsequently, the animals were exercised for 39 days in either running wheels or by swimming in a circular pool before starting a spatial acquisition task in a water maze. The MCAO running group found the platform significantly faster than the nonexercised MCAO group. This was not the case for the swimming group, whose performance did not differentiate from the nonexercised MCAO group. Progenitor cell survival in the dentate gyrus and pCREB levels were increased in the MCAO running wheel group compared to the control group. This suggests that different exercise types can affect cognitive recovery differently and that voluntary exercise initiated after the first postinjury week can induce functional recovery and help promote cell survival.

However, in another study by Piao et al. [31] starting exercise 1 week after injury did not produce similar results. Examining the timing effects of a 4-week running wheel regimen after controlled cortical impact injury (CCI), animals were exercised beginning either 1 week (“early”) or 5 weeks (“late”) postinjury. The study found that the late exercised CCI-group had a significantly reduced latency to find the platform in a spatial water maze learning task and better retention of the task compared to a nonexercised CCI-group. In a reversed platform test, a test of cognitive flexibility, they found that the late exercised animals showed a significant improvement compared to both the early exercised CCI-group and the nonexercised CCI-group. Retention of the reversed platform task was significantly better in the late exercised CCI-animals compared to the nonexercised CCI-group. There were no differences between the early initiated group and the nonexercised CCI-group on any of the above parameters. In a novel object recognition task, the late exercised CCI-animals spent significantly longer time exploring a new object than both the early exercised CCI-group and a nonexercised CCI-group, indicating improved short-term memory abilities; in fact their exploration time was at the level of uninjured, naïve animals. There were no group differences in locomotor activity in an open field test. In a tail suspension test, all CCI-groups showed increased immobility times regardless of exercise status, suggesting more pronounced behavioral despair due to injury. Furthermore, the late exercised CCI-group had a reduced lesion size compared to the early exercised CCI-group and the nonexercised CCI-group. There were time-dependent increases and decreases in different microglia activation markers: IL-1*β* levels (a proinflammatory marker) increased in the early exercise group in week 5 after CCI and levels reduced in the late exercise group in postinjury week 9 (both compared to the nonexercised CCI-group). There were also increased levels of IL-6 (a proinflammatory marker) and IL-10 (an anti-inflammatory marker) in the late exercise group in week 9. Cortical (ipsilateral) Galectin-3 and C1qB levels (microglial activation markers) were increased in the early exercised animals, while they were reduced in the late exercised group together with levels of gp91phox and p22phox (membrane components of NADPH oxidase enzyme). Late exercise increased hippocampal CREB gene expression, BDNF, and IGF-1 (insulin-like growth factor 1) levels and increased neurogenesis and cell survival in the late exercise group (but not in the early exercise group). The study concludes that the improved cognitive performance in the group subjected to late exercise is possibly due to a more optimal coordination/balance of microglia expression and increased growth factor levels.

Similar to studies initiating exercise immediately after ABI, starting a voluntary exercise paradigm one week after injury produces conflicting results, suggesting that the exercise type (voluntary versus forced) is not the only factor determining the efficacy of exercise.

As already mentioned, Griesbach et al. [39] found improved cognitive performance in animals exercised 14 days after injury. In a later study, Griesbach et al. [42] reproduced this finding. Animals exercised 14 days after lFPI acquired a spatial learning task in a water maze significantly faster than nonexercised lFPI animals. In addition, they reached six out of seven criterion scores (e.g., reaching a platform from 10 sec down to 4 sec) significantly faster than the nonexercised lFPI group. Exercise increased hippocampal levels of BDNF in all animals regardless of lesion status. However, mBDNF and CREB levels were higher in the lFPI exercised animals than in the lFPI control animals. This also held true for the exercised sham animals, who showed increased levels of Synapsin-I. When blocking trkB receptors in lFPI animals, the exercise-induced increase in mBDNF was reduced. The two studies by Griesbach et al. [39, 42] suggest that voluntary exercise started at a later stage (14 days) is beneficial for cognitive recovery, possibly through upregulation of BDNF and down-stream effectors of synaptic transmission.

Wong-Goodrich et al. [43] subjected female C57BL/6 mice to whole brain irradiation (5 Gy, single dose). The animals were then given access to running wheels outside of their home cages for 8–12 hours a day, starting 1 month after irradiation. Prior to initiation of exercise, all animals were tested in a spatial learning and retention test in a Barnes maze as well as a tail suspension test. After 6 weeks of wheel running, the animals were once again tested in the Barnes maze (both 3 and 4 months after irradiation) and in the tail suspension test (2.5 months after irradiation). Results from the first testing period in the Barnes maze (preexercise) showed no differences between the groups: the sham animals learned the task by day 2, the irradiated animals by day 3. There were no differences in retention as assessed by probe trials. The tests performed after exercise showed that irradiated, exercised animals had longer latency to complete the task on the first test-day compared to all other groups; however, by day 3 there were no differences. The irradiated, sedentary animals did not exhibit target quadrant preferences in retention trials (which they did during the first test period). However, the irradiated, exercised animals spent more time in the target quadrant, indicating improved memory. This picture was also seen on the test performed 4 months after irradiation. There were no differences between the groups in immobility times in the tail suspension test at any test point. Histology showed no differences in dentate gyrus size in any of the groups. Running elevated the number of hippocampal BrdU and NeuN positive cells (markers of newborn cells and mature neurons, resp.) in the irradiated animals. Levels of proinflammatory cytokines (tumor necrosis factor-*α* (TNF-*α*), interferon-*γ* (IFN-*γ*), and interleukin-6 (IL-6)) were elevated in the irradiated groups (compared to shams). Levels of IGF were increased in the irradiated, exercised animals compared to irradiated, sedentary animals. Levels of BDNF and VEGF (vascular endothelial growth factor) were decreased in all irradiated animals (both exercised and nonexercised). However, running partially restored VEGF-levels in the irradiated, exercised animals. The study shows that later-initiated voluntary running can prevent memory decline at a later stage in irradiation-exposed animals.

Showing similar positive recovery effects of late-initiated voluntary exercise after brain irradiation, Winocur et al. [44] irradiated adult rats at a single dose of 8 Gy. Twenty-five days after irradiation approximately half the animals were allowed to exercise in running wheels in their home cages. After two weeks of running, all animals were tested in a visual discrimination task in a water maze, followed by either a high-interference task (an unsolvable task) or a low-interference task (demanding no visual discrimination for task solution). Lastly, a retention test for the original discrimination task was performed. There were no differences between the groups in acquiring the visual discrimination task. In the retention task, the irradiated animals had the most errors; this was especially pronounced in the animals that had previously performed the high-interference task. Further analysis showed that irradiated, exercised animals in the high-interference group performed significantly better in the retention test than the irradiated, sedentary animals in the low-interference group. The exercised, sham animals performed better in the retention test than the sedentary sham animals regardless of interference group affiliation. Analysis of hippocampal DCX and ki67 positive neurons (neurogenesis markers) showed that irradiation decreased their levels, but running increased the levels in all exercised groups. The authors conclude that neurogenesis is a part of the mechanism that controls memory interference, as suppressing neurogenesis disrupts retention in the high-interference groups. However, this effect can be diminished by promoting neurogenesis through exercise.

While three studies found cognitive improvement in animals starting exercise 25 days after injury or later [31, 43, 44], Clark et al. [45] administered running wheel exercise between 114 and 142 days after gamma irradiation of the hippocampal area of both male and female C57BL/6J mice. They found that 54 days of wheel exercise did not have an effect on spatial learning and retention in a water maze in the gamma radiated group compared with a nonexercised radiated group. Running did, however, have a positive effect on sham operated animals. In a contextual fear conditioning test, running increased freezing time (indicating increased memory of a formerly presented painful stimulus); however, this was regardless of radiation status. In other words, in the spatial tasks no effects of exercise were found in the irradiated animals, yet running did improve performance in the conditioning task in all exercise groups. Running increased hippocampal neurogenesis regardless of lesion status. Exercise counteracted radiation-induced reductions in neurogenesis, neuronal differentiation, and glia cell levels.

Five studies [31, 39, 42–44] found positive effects of later initiated voluntary exercise on measures of spatial learning and retention. However, this was not the case in the study by Clark et al. [45], who waited 3-4 months with exercise administration. This opens the question whether there is a window of rehabilitation opportunity that closes after certain amount of time has passed. Other factors could also account for the conflicting results such as different injury types and duration of exercise. Interestingly, running affected performance positively in the fear conditioning task in the Clark et al. study, indicating that exercise effects can be task specific.

All in all, the above research shows a somewhat mixed picture of using voluntary exercise in cognitive rehabilitation after ABI. Later starting points (from 14-days post-injury) appear to have the most consistent effects on cognitive recovery. However, further research is needed to determine if exercise interventions can be administered too late to produce cognitive improvements. Moreover, caution should be taken in making general recommendations based on such a limited and methodologically diverse set of studies. The results indicate that the voluntary aspect of exercise is not the sole determinant of effect; other variables such as starting point and duration may also play a significant role.

### 3.2. Forced Exercise

Fourteen studies have looked into the effects of forced exercise on cognitive recovery after ABI.

Itoh et al. [46] subjected rats to CCI injury followed by a 7-day treadmill exercise regimen beginning one day after injury. In acquisition and retention of a spatial task in a water maze, the lesioned, exercised animals did significantly better than the lesioned, nonexercised animals; the former performing to the functional level of the sham animals. There was a significant reduction in lesion size in the exercised group compared to the controls. Additionally, there was a significant reduction in ssDNA immunopositive cells (a marker of apoptosis) around the damaged cortical area in the exercised group on postinjury days 1, 3, and 7, an increase in the number of NeuN positive cells, and a reduction in GFAP positive cells (marker for astrocytes) 7 days after TBI compared to the lesioned, nonexercised control group. This suggests that early initiated forced exercise can improve cognitive function while reducing apoptosis and impacting the glial scarring.

Cechetti et al. [47] looked at effects of both pre- and postinjury treadmill exercise in a bilateral common carotid artery occlusion (CCAO) rat model. The postinjury trained group started exercising 24 hours following surgery and continued for 12 weeks, 3 days a week. They found that all exercised groups, including the postinjury exercised group, did significantly better on three of the five testing days in acquisition of a spatial task in a water maze compared to a lesioned, nonexercised group. This pattern was also seen in a retention (probe trial) test and in a working memory test in a water maze. There were no differences between groups in levels of free radicals or SOD (superoxide dismutase, an antioxidant enzyme) levels. However, there were heightened hippocampal lipoperoxidation (evaluated by TBARS test) and thiol-levels (antioxidants) in the lesioned, nonexercised group compared to the other groups. Like the study by Itoh et al. [46], this study shows that early initiated forced exercise can positively affect cognitive recovery, potentially through reducing oxidative damage by regulation of antioxidant levels.

Shih et al. [48] subjected rats to right hemisphere MCAO. After 24 hours, the animals began exercising at either a low or a high-intensity (speed) on a treadmill for 14 days. They found that the lesioned, low-intensity group had shorter latencies on three out of the four testing days in a spatial learning task in a water maze (compared to the lesioned, high-intensity group and a lesioned, nonexercised control group). The low-intensity group also showed better retention than the control group. Furthermore, the low-intensity paradigm increased levels of hippocampal BDNF, Synapsin-I (contralaterally), and PSD-95 (membrane scaffolding protein) as well as the dendritic complexity (measured by Sholl analysis) and the number of dendritic spines compared to the control group. There were higher levels of corticosterone (stress-hormone) in the high-intensity group compared to the controls. The study is interesting as it investigates the effects of exercise intensity on cognitive measures. While both groups initiated exercise 24 hours after injury, only the low-intensity group showed positive cognitive effects concomitant with increases in plasticity-related proteins and dendrite development. Furthermore, the high-intensity group displayed higher levels of stress-hormone, which may have inhibited the efficacy of exercise.

In another study investigating the effects of different exercise intensities, Shen et al. [49] subjected rats to CCI immediately followed by two different intensities of treadmill exercise for 14 days. They found that the lesioned, low-intensity group performed better on two out of the four days of the acquisition part of a spatial task in a water maze compared to the high-intensity group and a lesioned, nonexercised control group. The low-intensity group also showed better retention than the control group. On a neurological deficit score all CCI animals did worse than the sham animals, but they all improved by day 6 post-TBI. BDNF and phosphorylated CREB measurements showed higher levels in the contralateral hippocampus in the low-intensity group compared to the control group. There were no differences in measurements of Synapsin-I and CREB in any of the groups.

While the above studies suggest that early forced exercise can promote cognitive recovery, these results are not unchallenged. Hicks et al. [50] found that animals exposed to lFPI and 18 days of treadmill exercise initiated the day following injury differed in neither spatial acquisition nor retention tasks in a water maze compared to a lesioned, nonexercised group. They saw no differences between groups in neuromotor scores. They found increased BDNF mRNA levels in CA1 and CA3 in the exercised, lesioned animals compared to lesioned, sedentary animals. There were no differences in hippocampal injury or cortical lesion volume between groups. However, the left neocortex (ipsilaterally to the injury) was significantly smaller than the right neocortex in the nonexercised, lesioned animals compared to the exercised, lesioned animals. These results are in contrast to many of the above studies, as they fail to find cognitive effects of early initiated forced exercise, but do find BDNF and some histological effects of exercise.

The findings of Hicks et al. [50] were echoed by Song et al. [51], who used photothrombosis to induce cerebral stroke in rats. One day after injury the animals were swim-exercised in a circular pool for 4 weeks (a total of 20 days), or given Acetyl-L-carnitine (ALC) injections, or both. They found no significant differences in any of their treatment groups compared to lesioned controls on acquisition of a spatial task in a water maze tested the first, second, and fourth week after injury. Hippocampal SOD-levels were increased in all treatment groups compared to the lesioned controls; these were significantly higher in the combined (exercise + ALC-injection) group in comparison to the other groups. MDA-levels (related to lipid peroxidation) were reduced in the treatment groups compared to the controls. Histologically, there were an increased number of cells in CA3 in the treatment groups compared to the controls.

Investigating emotional parameters, de Araujo et al. [52] exercised gerbils on treadmills either 12, 24, 48, or 72 hours after CCAO for 1 up to 3 days. In an open field, animals exercised 12 hours after injury showed a decreased number of field crossings and an increase in grooming (indicating increased anxiety and stereotyped behavior) compared to a nonlesioned, nonexercised group. There were no differences in any other groups. All CCAO animals showed a reduced time spent on a rotarod compared with the nonlesioned, nonexercised group. There were a decreased number of cells in CA1 and striatum in the group exercised after 12 hours compared to the group exercised after 24 hours. This study indicates that very early initiated short-duration exercise (12 hours after injury) can lead to increased anxiety-like behavior and cell death, while exercise starting 24 hours (or later) does not induce these emotional responses. Unfortunately, the experimental groups did not exercise the same amount. Exercise doses were decreased with later initiation points (down to a single 15 min session), making it difficult to decipher starting point effects from dose-related effects in the exercised groups.

Some studies have opted for exercise initiation two days after injury. After inflicting bilateral CCAO in gerbils, Sim et al. [53] found that treadmill exercise for 10 days resulted in longer latencies (i.e., better short-term memory for a noxious stimulus) in a step-down avoidance task than in a nonexercised, lesioned group. They also found reduced levels of TUNEL positive and Caspase-3 positive cells (markers for apoptosis) in the lesioned, exercise group compared with the lesioned, nonexercised group. Cell proliferation was increased in the nonexercised, lesioned group and the exercised, sham group, but not in the lesioned, exercise group. The authors hypothesized that this finding might be due to reduced cell death in the exercised group, reflected in less cell proliferation. In a later experiment, using the same injury model but a longer exercise regimen (4 weeks, starting on the first postinjury day), Sim et al. [54] found that the lesioned, exercised animals did better than the nonexercised lesioned group in the step-down avoidance task. The exercised, lesioned group presented with fewer TUNEL and Caspase-3 positive cells than the nonexercised, lesioned group. The studies indicate that exercise might protect the brain from neuronal cell death, which could play a part in the functional recovery. Interestingly, this finding goes for both a shorter and longer duration exercise paradigm at the same running speed.

Chen et al. [55] exposed rats to hippocampal injury via unilateral kainic acid injection to the CA1 area. Starting on the second postinjury day, the animals were exercised in a motorized running wheel for seven consecutive days at one of three different intensities: light, moderate, and heavy. Exercise took place twice a day (morning and afternoon) for 30 minutes. The animals were then tested in a conditioning (pain-avoidance) learning task in a Y-maze for one session of 20 trials. The study found that lesioned animals that had been exercised at moderate intensity performed significantly better in the learning task than nonexercised, lesioned animals, as well as showing significantly higher numbers of BrdU positive cells. There were no learning or BrdU staining differences between the other lesioned, exercised groups and the nonexercised, lesioned animals. Furthermore, a positive correlation between learning and BrdU positive labelled cells in the dentate gyrus was found, indicating that neurogenesis may have supported the functional recovery.

Positive recovery effects of second day postinjury initiation were also found by Kim et al. [56]. Using electromagnetic contusion in rats followed by 10 days of treadmill exercise, they found that lesioned, exercised animals had shorter latency times in a step-down avoidance test than a lesioned, nonexercised group, indicating better (short-term) memory for a noxious stimulus in the exercised group. Measurements of hippocampal DNA fragmentation (a marker for apoptosis), Caspase-3, and Bax (pro-apoptosis molecules) showed reduced levels in the lesioned, exercised group compared to the lesioned, nonexercised group. Levels of Blcl2 (antiapoptosis molecules) were increased in the lesioned, exercised group compared to the control group. There were no differences in corticosterone levels between the groups. Besides improvement in short-term memory, the study, like those by Sim et al. [53, 54], shows effects on markers of apoptosis further supporting the assumption that enhanced functional recovery after exercise could be mediated by regulation of neuronal cell death mechanisms.

Chen et al. [57] compared the timing of treadmill exercise initiated two days (early, for either 7 or 14 days) or nine days after injury (late, for 7 days) in a closed head injury mouse model. In an object recognition task, they found that the early initiated groups spent significantly more time exploring a new object compared to a lesioned, nonexercised group indicating better memory for the previously encountered object. The late initiated group and the nonexercised, lesioned group spent less time exploring the new object than the sham animals. Furthermore, early exercise hindered progressive cell loss in the cortex and the hippocampus to a larger extent than in the late exercised group. Early exercise boosted neurite regeneration in the early postinjury stages, but late exercise only hindered later stage cell loss. Early exercise for 14 days restored the lesion-induced reduction in BDNF and MKP-1 (an anti-inflammation marker). When animals were given triptolide (a MKP-1 synthesis inhibitor) neither this nor cognitive recovery was seen; however, there were positive effects on neuronal loss and neuroinflammation. These findings show that different starting points can generate different outcomes in short-term memory, cell survival, and plasticity-related protein levels.

Starting exercise on the fourth day after injury, Shimada et al. [58] subjected rats to left MCAO followed by one of two different treadmill intensities (low or high) for 28 days. In both an object recognition and an object location task, the low-intensity group spent more time exploring the novel object/newly placed object than the lesioned, nonexercised control group. The high-intensity group explored less than the low-intensity group. In a passive avoidance test, both exercise groups showed longer latencies than the controls, indicating that exercise resulted in better memory for noxious stimuli. An open field analysis did not reveal any locomotor differences between the groups. Exercise reduced lesion size, but there were no differences between the intensity groups. Both intensity groups had increased number of neurons in the dentate gyrus compared with the controls and the shams; this was higher in the ipsilateral dentate gyrus in the low-intensity group versus the high-intensity group. In the ipsilateral dentate gyrus, MAP-2 levels (microtubule-associated protein 2) were increased in the low-intensity group compared to the controls. MAP-2 was lower in the high-intensity group compared with the low-intensity group and the shams ipsilaterally in all examined hippocampal areas. Contralaterally, the levels were lower in CA1 and CA3 in the high-intensity group than in the low-intensity group. The findings of this study echo the findings of Shih et al., Shen et al., and Chen et al. [48, 49, 55] underlining the potential differential effects of varying exercise intensities in the early stages of recovery. As in the studies by Shih et al. and Shen et al., this study shows that low-intensity forced exercise is able to produce cognitive recovery effects after ABI; however, in this study there were also positive effects of high-intensity exercise on one of the cognitive parameters (passive avoidance).

Two studies have begun forced exercise 1 week after ABI or later. The previously mentioned study by Luo et al. [41] compared a forced exercise protocol (swimming) with a voluntary exercise protocol starting one week after MCAO. They found no cognitive effects of the forced exercise paradigm. Chen et al. [57] (see above) did not find any effects of exercise starting 9 days after injury on cognitive parameters. This opens the question as to whether there exists a window of opportunity for rehabilitation via forced exercise that closes after a certain time point. Compared to what appears to be the case regarding voluntary exercise, this window may be substantially smaller.

The studies of forced exercise, like those of voluntary exercise, show a somewhat conflicting, pattern of outcome. Though only one study shows detrimental effects (in one group) on anxiety-related behavior, the above studies generally show that especially early forced exercise can lead to improvement in animals exposed to low or moderate intensity exercise. One may therefore ask whether exercise needs to be maintained at a certain intensity level in order to produce cognitive gains. Neither of the two studies using swimming exercise produced cognitive recovery effects. However, more studies are needed to determine whether this is a result of the type of exercise or protocol related issues. Unfortunately, only two studies investigated effects of exercise starting later than a week, leaving us with limited knowledge about the effects of forced exercise initiated at a later stage.

## 4. Sooner or Later?

As already described, the above research varies in the time points of exercise initiation. Of the 22 studies included in this overview, we find that 16 studies had experimental groups starting exercise from postinjury days 0–4, while eight studies had experimental groups starting exercising at the earliest from postinjury day 7.

Examining common traits or dissimilarities of the early intervention groups with positive or no effects does not render a clear picture. Almost all early initiation studies with positive effects of exercise on cognition use forced exercise paradigms. However, as early initiation voluntary exercise studies are much fewer in number, this might be a paradigm bias. Moreover, the studies vary on most parameters including types of injury and animal as well as exercise duration and intensity.

The three studies showing groups with adverse effects [39, 40, 52] started exercising the animals immediately after injury, that is, within the first 24 hours. They also had fairly short duration exercise protocols (3 or 7 days). This indicates that very acute, relatively short duration exercise can induce unwanted effects. However, positive cognitive outcomes using very early exercise have also been reported [38] (see above).

Later initiation studies are fewer and with starting points spanning from 1 week to almost 4 months after injury; it is difficult to obtain a coherent picture. Studies with groups starting 7 to 9 days after injury [31, 41, 57] showed either positive and/or no effects, and a 14 day postinjury start showed cognitive improvement effects in two studies [39, 42]. Starting at even later time points showed some variability: starting 25–30 days post-ABI induced positive effects in three studies [31, 43, 44], while an approximately 4-month postinjury start generated both an improvement and no effects depending on the cognitive measure [45]. Thus it would appear that later initiated exercise, in most cases, can promote cognitive recovery. However, once again, there are considerable methodological variations between the studies.

It is quite surprising that we know relatively little about the cognitive effects of exercise starting relatively late post-TBI. In clinical rehabilitation settings exercise is often initiated in later recuperation stages, when patients are stabilized and able to perform physical activities. It therefore seems clinically relevant to further investigate the potential effects of late-initiation exercise.

## 5. Easy Does It?

Exercise dose encompasses many variables including total length of intervention (how many days), session duration (how many minutes), distribution (how often), distance moved (how far), and intensity (how fast).

In the 22 studies included in this review, the total length of intervention varied considerably, ranging from 1 day to almost 4 months. Regarding individual session durations, most of the voluntary exercise studies gave the animals unlimited access to the running wheels, that is, 24 hour access. The forced exercise paradigm sessions lasted between 5 min and 1 hour; eight of those studies used 30 min session durations. By and large, the animals exercised/or had access to exercise apparatuses on a daily basis throughout the intervention period except in two studies that distributed the intervention somewhat differently [47, 51], as well as one study that exercised animals twice daily [55].

Average group distances and/or intensities are not stated in all studies (see Table 1). This information is provided in five voluntary paradigms and 12 forced paradigms. Intensities are mostly reported in meters exercised pr. minute and often vary within individual exercise sessions or over days/weeks. In some cases, exercise duration (number of minutes) is increased over a period of days. While such graduation of intensity or duration of exercise might in itself be an important rehabilitative factor, the individual protocols vary too much for meaningful comparisons to be carried out. When calculating mean daily/session distances over the total duration of exercise, they range between 97.2 m and 8.4 km. Such a wide variation is also found in the total exercise distances over time (i.e., total distance over all exercise days/sessions) that range from 150 m to 313.2 km.

Four studies explicitly examined the cognitive effects of different exercise intensities [48, 49, 55, 58]. Interestingly, all of these studies found that the low or moderate exercise intensity groups produced positive results, while the higher intensity groups did not produce any results (or only produced results in one test [58]) (see above). This indicates that intensity is indeed an important factor when using exercise as a cognitive rehabilitation tool. While it would appear that average doses up to around 250 m daily in many cases produce positive results [31, 48, 49, 53, 54, 56, 58], this is not always the case [31, 52, 55]. The picture becomes more blurred when using higher daily doses. In the studies specifically looking into exercise intensities, daily doses exceeding an average of 320 m daily did not produce cognitive results in three of the studies. It did, however, produce positive results in one study [55]. In other cases [43, 44, 46, 47, 57] average session distances of 320 m and above improved cognitive recovery, but this was in some cases contingent upon other variables such as starting point. In some studies, doses exceeding 320 m daily did not produce any results on the spatial tasks [45, 50] or had detrimental effects [40].

Interestingly, in the case of the Chen et al. study [55], the moderate exercise group (that showed positive recovery effects) ran 180 m twice daily, making the individual exercise trials fall below the 320 m mark (but the total daily running distance was slightly above). However, their heavy intensity group ran 324 m twice daily (to a total of 648 m) and did not show recovery effects. One may therefore ask whether total running distances are a good dose measure, or whether the intensity of individual training trials are of more importance for cognitive recovery. Although an unresolved matter, this could be another explanatory factor for the differential results in studies examining voluntary running effects, where intensity and duration of individual running bouts are not experimentally controlled.

All in all, the substantial variations in exercise protocols among the studies make it difficult to make general dose recommendations. While it does appear that dose, duration, and intensity are important factors for cognitive recovery, more systematic research looking into these aspects and how they interact with other variables such as starting point is needed to elucidate this further.

## 6. Post-ABI Exercise and Brain-Derived Neurotrophic Factor (BDNF)

While many neural mechanisms behind the effects of exercise are being investigated, special attention has been given to neurotrophic factors, in particular BDNF. BDNF is highly expressed in the cortex and hippocampus and is involved in many neural processes including neuronal differentiation and survival, as well as axonal path-finding [59]. Furthermore, the relationship between forced exercise and stress-hormone levels has garnered considerable interest. In the following these topics will be investigated further in relation to exercise type, timing, and intensity.

### 6.1. Exercise Type, BDNF, and Stress-Hormone

In relation to exercise type, a special focus has been placed on the connection between exercise and the release of stress-hormones, as forced exercise is believed to be more stressful than voluntary exercise. However, studies dealing with this topic show somewhat inconsistent results. Griesbach et al. [60] found that early stage postinjury forced exercise elevated corticosterone and ACTH levels in lFPI animals. This was not the case in a group exercised in a voluntary paradigm. Neither exercise regimens elevated BDNF-levels. In another experiment starting exercise at a later stage, Griesbach et al. [61] found that forced exercise stimulated the corticotrophic axis in all animals. BDNF-levels were unaffected by forced exercise, yet they were elevated in all rats exposed to voluntary exercise. In two other studies [42, 62] the same lab also found an increase in BDNF-levels as a result of voluntary exercise. Similarly, Ke et al. [20] found that voluntary exercise improved motor function and elevated BDNF-levels, an effect not seen in the group exposed to forced exercise, although these animals did present higher levels of corticosterone. Wong-Goodrich et al. [43] did not see any exercise-related BDNF-changes in their late voluntary paradigm; however, they did find that the intervention improved cognition in their irradiated animals.

Several studies using forced exercise after TBI have found BDNF-elevations [21, 57, 63–65], indicating that forced exercise paradigms can increase BDNF-levels after injury. Using both forced and voluntary exercise, Ploughman et al. [66] found that corticosterone levels were elevated in all exercise groups but were highest in animals exposed to forced exercise running at greater speed or duration. Exercise did not increase BDNF, IGF-1, or Synapsin-I in the ischemic hemisphere. Furthermore, they found that voluntary exercise decreased serum levels of IGF-1 and increased hippocampal levels of IGF-1 in the ischemic hemisphere. Shih et al. [48] (see above) also found corticosterone elevations in their high-intensity group. However, in the study by Kim et al. [56] (see above), no differences in stress-hormone levels were found between the treadmill exercised and nonexercised groups. Ploughman et al. [67] found that forced exercise created a rapid, but more short-lived BDNF-increase compared to voluntary exercise. The group exposed to forced exercise also showed increased levels of corticosterone in several brain regions.

Thus, it appears that forced exercise does lead to elevated stress-hormone levels. When it comes to impact on BDNF-levels, the picture is more unclear. It seems that the type of exercise (voluntary or forced) cannot solely account for variation in neurotrophic factor levels, but other factors such as timing and intensity are also key players. How stress-hormones and neuroplasticity-related proteins are affected by exercise, how they interact, and, importantly, what consequences this has for functional recovery remain to be resolved. As discussed above, though the efficacy of forced exercise on cognitive parameters is inconclusive, detrimental effects on cognition are practically unseen. This poses the question of whether elevations in stress-hormones during physical activity are necessarily harmful when it comes to the recovery of cognitive functions.

### 6.2. Exercise Starting Point and BDNF Responses

Like exercise type, some research indicates that starting point affects BDNF-levels after ABI. Early exercise initiation (defined here from day 0–6 postinjury) has been shown to elevate BDNF-levels in several studies [21, 50, 57, 62, 63, 65]; in some cases this elevation is also dependent upon exercise intensity [48, 49] (see below) or type of exercise [20, 67] (see above). In other cases, early exercise did not affect BDNF-levels [38, 39, 60]. Later post-ABI exercise (defined here from postinjury day 7 and onwards) has also been shown to produce BDNF-elevations [31, 39, 42], in some cases this is dependent on type of exercise [60] or injury severity [68]. A few studies have found that later initiated exercise did not produce BDNF-elevations [31, 43, 57].

These studies indicate that both early and later initiated exercise regimens can increase BDNF-levels in some cases. Whether such BDNF-elevations are part of the neural processes mediating cognitive recovery is still unclear. Griesbach et al. [39] did not find BDNF-elevations after early initiated exercise and this group also showed delayed learning. Wu et al. [38] found no BDNF-effects either but did see improvements in their cognitive measure after early initiated exercise. Reversely, Hicks et al. [50] found BDNF-elevations after early initiation, but no cognitive effects. Chen et al. [57] found elevated BDNF-levels (in their early initiated group running for 14 days) as well as a cognitive improvement. Shih et al. [48] and Shen et al. [49] also found both BDNF-elevations and cognitive improvements (in their low-intensity running groups).

Initiating exercise at later points, Griesbach et al. [39, 42] found BDNF-elevations and concomitant cognitive improvement. The same holds for the study by Piao et al. [31]; however only in one of their two (late) exercised groups. Wong-Goodrich et al. [43] found no exercise-related BDNF-level changes in their irradiated animals; they did, however, find a cognitive improvement.

It seems that the relationship between BDNF-responses and cognitive recovery outcome at different exercise initiation points is still largely unresolved. Currently, there are a limited number of studies investigating this, underlining a need for additional research.

### 6.3. BDNF and Post-ABI Exercise Intensity

Not many studies have investigated the relationship between BDNF-levels and exercise intensity in post-ABI exercise. Shih et al. [48] and Shen et al. [49] found hippocampal BDNF-elevations (contralaterally) in their low-intensity exercise groups concomitant with cognitive improvement. In a study by Ploughman et al. [66], rats were subjected to focal stroke using endothelin-I. After 4 days of recovery, the animals were given either a 30 min or a 60 min walk in a motorized running wheel (both 11 m/min), a 30 min run in a motorized running wheel (14 m/min), or a 12-hour voluntary run in a (nonmotorized) running wheel. The animals in the 30 min motorized walking group and the voluntary running group had increased hippocampal BDNF-levels (in the noninjured hemisphere) compared to noninjured, nonexercised animals. Furthermore, the 30 min walking group showed increased BDNF-levels in the intact sensorimotor cortex compared to the 60 min walking group and nonexercised animals. Placed together, these studies indicate that exercise of a lower intensity can increase BDNF-levels in areas contralaterally to the inflicted injury. However, intensity and duration of intervention (and thereby total distance run) vary between the studies, restricting what overall information can be derived regarding the relationship between BDNF and post-ABI intensity parameters.

## 7. General Considerations

The above studies provide some information as to the effects of exercise on cognition in the brain injured individual. They also stress some of the parameters that are important for the efficiency of this intervention. However, there are still many unresolved issues.

Voluntary and forced exercise paradigms vary on parameters of choice of movement and, potentially, level of stress-hormone activation. The studies included in this review also reveal other differences between the two exercise paradigms. Most of the voluntary paradigms allow animals access to the exercise apparatus in their home environment, while the forced paradigms require moving and handling of the animals to initiate (and sometimes prompt) exercise. Whether such environmental differences can affect the outcome in terms of cognitive recovery will have to be clarified in the future.

In all but two of the studies using voluntary exercise, animals were housed individually either permanently or during intervention. Most of the forced exercise studies do not report housing conditions; however those that do have animals pair or group housed. Some studies have looked into the effects of social deprivation and exercise in animals. Stranahan et al. [69] found that both single and group housed male rats had corticosterone elevations due to running. However, only group housed animals also presented increased neurogenesis induced by running. When exposing these animals to additional stressors, the socially isolated animals showed decreased neurogenesis compared to the controls. In another study using female rats, Leasure and Decker [70] found that social isolation suppressed the cell-proliferation effects of exercise that were seen in group housed animals. Furthermore, there was a correlation between BrdU+ cells and the running distance in the group housed animals, but not in the single housed animals. In a study looking into the emotional effects of housing, Berry et al. [71] found that single housing triggered anxiety and depression-like behaviors in the animals, increased HPA-axis reactivity, and reduced BDNF-levels. Such findings indicate that housing-paradigms (and animal gender) can influence the effects of exercise as well as emotional reactivity. Whether single housing would also influence cognitive performance in animals subjected to ABI remains unknown. It is therefore relevant to investigate whether cognitive effects in exercise studies using single housing are related to the exercise intervention per se, boredom-factors due to isolation, or other variables.

Furthermore, there are considerable differences in relation to exercise dose and duration. Animals in the voluntary paradigms have 24-hour access to the exercise apparatuses (except in one study) and can administer their treatment when they choose and in the intensity and duration that they prefer. This is a marked difference from forced exercise paradigms that mainly offer single exercise bouts of limited duration (up to 1 hour) under controlled running speeds. These differences underline that paradigms of voluntary and forced exercise vary on many variables that can affect the cognitive (and neural) outcome.

Epidemiological research shows that premenopausal women have decreased risk of stroke compared to age-matched males as well as to postmenopausal women [72]. Animal studies have shown that female hormones regulate and protect against a variety of pathological processes associated with stroke [73]. Both estrogen and progesterone have been shown to have neuroprotective effects after stroke in animal models [73, 74]. This indicates that gender-specific hormonal environments can influence the recovery outcome after brain injury. However, all but two of the studies discussed in this review use male animals (see Table 1), leaving us with very limited data about the effects of exercise in the traumatized female brain.

The type and severity of injuries in the above studies are quite different. Some types of injury cause more focal tissue damage; others are more wide-spread and diffuse in nature. Some injuries are unilateral, while some affect both hemispheres. The studies using cerebral ischemia models inhibit blood flow for varied time periods. The studies using traumatic brain injury models (i.e., an external force afflicting the brain) use different techniques, impact velocities, and depths of compression. The models inducing injury by irradiation use different doses and afflict different cerebral areas. As different types of brain injury and injury severities can cause different injury patterns, both in terms of tissue responses as well as their spatial and temporal occurrence [75], this can also affect the efficacy of the employed exercise protocols.

Another issue relates to the genetic make-up of the experimental animals. Much research has shown that the same brain injury method can induce significantly different cerebral (and behavioral) responses depending on the rodent strain/stock used [76–90]. Even animals of the same stock, but purchased from different breeders, have been shown to differ in their cerebral responses when exposed to the same injury [91–93]. Thus, strain/stock choice is an important factor to take into account when assessing brain injury outcomes as well as the efficacy of treatment interventions. In the 22 studies included in this review, six different rodent strains/stocks were used (see Table 1). However, due to the considerable procedural differences in performing “the same” brain injury (see above) as well as substantial interstudy variations in the exercise protocols and outcome measures, meaningful comparisons of the studies on the basis of strain are very difficult to make. Further research is needed to elucidate the effects of strain on post-ABI exercise on cognitive recovery.

The applied cognitive tests are generally brief, limiting our knowledge to mainly short-term learning effects. Potential long-term effects of exercise have not been examined in any of the studies, leaving us with little knowledge as to whether the observed cognitive effects are lasting or transient. Furthermore, the majority of studies use tests that motivate learning through avoidance, that is, the ability to avoid an unwanted stimulus (escaping water or a previously presented painful stimulus). Testing animals in nonavoidance based tasks would help to clarify whether the outcome is related to the treatment or the method of testing.

Another discussion related to the cognitive tests pertains to the individual test protocols and setups. While many of the studies used spatial acquisition tasks in a water maze, the individual testing protocols were very varied, in terms of both number of acquisition trials and sessions. Such differences could potentially affect the learning outcome if some animals were to be trained more intensively than others [94]. Furthermore, the visual surroundings when performing spatial acquisition tasks (i.e., the number and salience of visual cues as well as their distance to the animals) have been shown to be of importance for both the neural substrate and cognitive mechanisms of task solution in rodents [95–97]. It is generally taken for granted that different cognitive tasks reflect different neural substrates and cognitive mechanisms. However, within what is generally considered the same cognitive tasks, various experimental and/or test setups can also vary with respect to the underlying neural and cognitive mechanisms [98, 99]. Consequently, what may superficially appear to be the same cognitive test may result in different cognitive recovery effects of a given exercise protocol; even minor variations in experimental setups can be essential. Thus, it appears that research in this area would benefit greatly from more homogenous use of cognitive tests/setups to facilitate comparisons between labs and help eliminate test protocol differences as a source of variation when assessing the effects of exercise on cognition.

Postinjury depression and anxiety are common after brain injury [100]. As already mentioned, exercise is often used in the treatment of depression and anxiety-related disorders.

It is known that depression can lead to cognitive impairment. However, whether these impairments are primarily psychosocially or neurobiologically founded, transient or enduring, is still debated [101]. Though some of the above studies have included tests of emotional behavior in their experimental protocols, we still know very little about how post-TBI exercise affects emotional states, and how this potentially affects cognitive performance. Knowing more about the relationship between injury-related emotional and cognitive problems will help to further clarify when (and in what way) exercise promotes cognitive recovery after ABI.

## 8. Conclusion

In this review we have examined the effects of exercise on cognitive measures after acquired brain injury in animal models. Although there is cause for optimism in using exercise as a rehabilitation tool in the treatment of cognitive sequelae after ABI, research in this area is still fairly limited. Overall, there is evidence that exercise in some cases can improve cognitive recovery. However, what distinguishes these cases from others that do not produce effects (or have adverse effects) remains unclear. Considerable variations in models and experimental protocols, including differences in animal strains, injury type, exercise type, post-injury starting point, dose-related differences, and cognitive measures, should presently warrant caution in making general protocol recommendations. More research is needed to clarify these issues as well as the potential long-term effects of postinjury exercise.

## Figures and Tables

**Table 1:** Summary of exercise protocols and postexercise cognitive/emotional and cerebral changes in ABI animal models.

Reference	ABI model	Animal, gender (age/weight)	Exercise type	Intervention start^*∗*^	Dose parameters	Mean daily exercise distance	Cognitive/emotional effects	Cerebral changes
de Araujo et al. [52]	CCAO	Mongolian gerbils, male (60–80 g)	Treadmill	Early (12, 24, 48, or 72 hours postinjury)	Max. 3 days (4 sessions), min. 1 day (1 session), 15 min/sess. Speed: 10 m/min	150 m	CCAO + EX after 12 hours showed a decreased number of field crossings and an increase in grooming in an open field test compared to sham + SED. No other group differences	CCAO + EX after 12 hours: number of cells ↓ in CA1 + striatum compared to CCAO + EX after 24 hours

Cechetti et al. [47]	CCAO	Wistar rats, male (3 months)	Treadmill	Early (24 hours postinjury^†^)	12 weeks, 3 times/week, 20 min/session. Weeks 1-2: 12 m/min for 3 min, 24 m/min for 4 min, 36 m/min for 6 min, 24 m/min for 4 min, and 12 m/min for 3 min; weeks 3–6, at 24 m/min for 4 min, 36 m/min for 12 min, and 24 m/min for 4 min; weeks 7–10: 24 m/min for 2 min, 36 m/min for 16 min, and 24 m/min for 2 min: weeks 11-12: 36 m/min for 2 min, 48 m/min for 16 min, and 36 m/min for 2 min.	480 m/session up to 912 m/session (graded protocol)	All EX groups did significantly better in a spatial acquisition and retention task as well as a working memory task than CCAO + SED	No differences between groups in levels of free radicals or SOD. CCAO + SED: hippocampal lipoperoxidation and thiol-levels ↑ compared to the other groups

Chen et al. [55]	NTI	Sprague-Dawley rats (12–14 weeks)	Motorized running wheel	Early (2nd postinjury day)	7 consecutive days, 30 min twice daily. LowEX: 3 m/min for 10 min., 4.2 m/min for 10 min, and 5.4 m/min for 10 min. ModEX: 4.8 m/min for 10 min, 6 m/min for 10 min, and 7.2 m/min for 10 min. HighEX: 9.6 m/min for 10 min, 10.8 m/min for 10 min, and 12 m/min for 10 min	NTI + LowtEX: 252 m; NTI + ModEX: 360 m; NTI + HighEX: 648 m	NTI + ModEX showed significantly better acquisition of conditioning task in Y-maze than NTI + SED. No acquisition differences between NTI + LowEx and NTI + SED or NTI + HighEx and NTI + SED	BrdU-positive cells in dentate gyrus ↑ in NTI + ModEX compared to NTI + SED. No BrdU-staining differences between other NTI + exercise intensity groups and NTI + SED. Positive correlation between acquisition and BrdU-positive cells

Chen et al. [57]	CHI	ICR mice, male (7 weeks)	Treadmill	Early + late (2 or 9 days postinjury)	7 or 14 days (early) or 7 days (late). 1 hour/daily. Speed: 9 m/min progressively increased to 13.5 m/min	Between 540 m and 810 m (graded protocol)	CHI + earlyEX spent significantly more time exploring new object in an object recognition task than CHI + SED. CHI + lateEX and CHI + SED had less time exploration time with the new object than sham animals	EarlyEX hindered progressive cell loss in the cortex and hippocampus more than lateEX. EarlyEX ↑ neurite regeneration in the early postinjury stages, lateEX hindered later stage cell loss. EarlyEX for 14 days restored lesion-induced reduction in BDNF and MKP-1

Clark et al. [45]	IRR	C57BL/6J mice, male + female (min. 50 days)	Running wheel	Late (114/142 days postinjury)	54 days total, 24-hour daily access	IRR + EX: 5.8 km; sham + EX: 5.7 km	No differences in spatial learning + retention between IRR + EX and IRR + SED. Spatial learning and retention improved in sham + EX. Exercise improved retention in a contextual fear conditioning test	Exercise ↑ hippocampal neurogenesis. Exercise counteracted radiation-induced reductions in neurogenesis, neuronal differentiation, and glia cell levels

Crane et al. [40]	CCI	Long Evans rats, male (ca. 50 days)	Running wheel	Early (immediately postinjury)	7 days	7-8 km	CCI + EX performed significantly worse in complex stop-signal reaction time task for the first five test days compared CCI + SED and sham groups. After a week of testing, CCI + EX returned to baseline levels	CCI + EX: GFAB and IBA1 positive cells ↑ in the cortex and hippocampus, respectively; all CCI-animals: DAP1 positive cells ↓ in the cortex, hippocampus, mediodorsal nucleus and corpus callosum compared to sham + SED

Griesbach et al. [39]	FPI	Sprague-Dawley rats, male (ca. 312 g)	Running wheel	Early + late (0 or 14 days postinjury)	7 days total, 24-hour daily access. Wheel resistance: 100 g	N/A (mean ranges of nightly running distances between approximately 300 m and approximately 3400 m)	FPI + earlyEX performed significantly worse on a spatial acquisition task than all other groups. FPI + lateEX performed to the level of the sham operated animals. All FPI animals performed worse than noninjured animals on retention task	FPI + lateEX and sham groups: hippocampal pCREB and BDNF ↑. FPI + earlyEX: Synapsin-I and CREB ↓

Griesbach et al. [42]	FPI	Sprague-Dawley rats, male (ca. 265 g)	Running wheel	Late (14 days postinjury)	7 days total, 24-hour daily access. Wheel resistance: 100 g	N/A	FPI + EX acquired a spatial learning task and reached six criterium scores significantly faster than FPI + SED	Exercise ↑ hippocampal levels of BDNF. FPI + EX: mBDNF and CREB ↑ compared to FPI + SED. Sham + EX: Synapsin-I ↑. When blocking trk-B receptors: mBDNF ↓ in FPI + EX

Hicks et al. [50]	FPI	Sprague-Dawley rats, male (360–410 g)	Treadmill	Early (1 day postinjury)	18 days, 11.3 m/min. Day 1: 5 min, increased by 5 min daily for 14 days until reaching 60 min. After this running 60 min again for 4 days. Inclination: 6°	56.5 m/daily up to 678 m/daily (time graded protocol)	No differences in spatial acquisition or retention between FPI + EX and FPI + SED	BDNF mRNA levels in CA1 and CA3 ↑ in FPI + EX compared to FPI + SED. No group differences in hippocampal injury or cortical lesion volume. Left neocortex < right neocortex in FPI + SED compared to FPI + EX

Itoh et al. [46]	CCI	Wistar rats, male (10 weeks)	Treadmill	Early (1 day postinjury)	7 days, 30 min/day. Speed: 22 m/min	660 m	CCI + EX did significantly better in a spatial acquisition and retention task than CCI + SED	Lesion size ↓ in CCI + EX compared to CCI + SED. ssDNA immunopositive cells around the damaged cortical area ↓ in CCI + EX (postinjury days 1, 3, and 7), number of NeuN positive cells ↑ and GFAP positive cells ↓ (postinjury day 7) compared to CCI + SED

Kim et al. [56]	CCI	Sprague-Dawley rats, male (7 weeks)	Treadmill	Early (2 days postinjury)	10 days, 30 min/day. Speed: 2 m/min for 5 min, 5 m/min for 5 min, and 8 m/min for 20 min	195 m (graded protocol)	CCI + EX had shorter latency times in a step-down avoidance test than CCI + SED	Hippocampal DNA fragmentation, Caspase-3, and Bax ↓ in CCI + EX compared to CCI + SED. Levels of Blcl2 ↑ in CCI + EX compared to CCI + SED. No group differences in corticosterone levels

Luo et al. [41]	MCAO	C57BL/6 mice, male (25–30 g)	Running wheel + swimming	Late (1 week postinjury)	RW: 43 days total, 24-hour daily access. SWIM: 43 days total, 2 trials/day, and 60 sec/trial	N/A	MCAO + RW had significantly shorter latency time to find the platform in a water maze task compared to MCAO + SED. No differences between MCAO + SWIM and MCAO + SED	Dentate gyrus progenitor cell survival and pCREB levels ↑ in MCAO + RW compared to control group

Piao et al. [31]	CCI	C57BL/6 mice, male (10 weeks)	Running wheel	Late (1 week or 5 weeks postinjury)	4 weeks total, 24-hour daily acces.	103.2 m (1 week), 97.2 m (5 weeks).	CCI + lateEX performed significantly better in a spatial acquisition and retention task than CCI + SED. CCI + lateEX showed significant improvement in a cognitive flexibility task compared to CCI + earlyEX and CCI + SED. Retention of the flexibility task was significantly better in CCI + lateEX compared to CCI + SED. In a novel object recognition task, CCI + lateEX spent significantly longer time exploring a new object than CCI + earlyEX and CCI + SED. No locomotor differences in an open field test. In a tail suspension test, all CCI groups had increased immobility times	CCI + lateEX: ↓ lesion size compared to CCI + earlyEX and CCI + SED. IL-1*β* levels ↑ in CCI + earlyEX (week 5) and ↓ in CCI + lateEX (week 9) compared to CCI + SED. IL-6 and IL-10 ↑ CCI + lateEX (week 9). Cortical Galectin-3 and C1qB levels ↑ increased in CCI + earlyEX, but ↓ in CCI + lateEX. Gp91phox and p22phox ↓ in CCI + lateEX. Hippocampal CREB gene expression, BDNF, IGF-1, neurogenesis, and cell survival ↑ in CCI + lateEX

Shen et al. [49]	CCI	Sprague-Dawley rats, male (250–270 g)	Treadmill	Early (24 hours postinjury)	14 days, 30 min/day. LowEX: week 1: 3 m/min; week 2: 3 m/min for 5 min, 5 m/min for 5 min, and 8 m/min for 20 min. HighEX: Day 1: 3 m/min; day 2: 3 m/min for 10 min, 6 m/min for 10 min, and 9 m/min for 10 min; day 3: 6 m/min for 10 min, 9 m/min for 10 min, and 12 m/min for 10 min; day 4–14: 12 m/min	Week 1: 90 m, week 2: 200 m (lowEW). Day 1: 90 m, day 2: 180 m, day 3: 270 m, day 4–14: 360 m (highEX) (graded protocols)	CCI + lowEX performed better on a spatial acquisition task compared to CCI + highEX and CCI + SED. CCI + lowEX showed better task retention than CCI + SED	Contralateral hippocampal BDNF and pCREB ↑ in CCI + lowEX compared to CCI + SED. No group differences in levels of Synapsin-I and CREB

Shih et al. [48]	MCAO	Sprague-Dawley rats, male (8 weeks)	Treadmill	Early (24 hours postinjury)	14 days, 30 min/day. LowEX: 8 m/min, highEX: 20 m/min	240 m (lowEX); 600 m (highEX)	MCAO + lowEX performed better in a spatial acquisition than the two other groups and showed better retention than MCAO + SED	Hippocampal BDNF, Synapsin-I (contralaterally), PSD-95, dendritic complexity and dendrite spines ↑ in MCAO + lowEX compared to MCAO + SED. Corticosterone levels ↑ in MCAO + highEX compared to MCAO + SED

Shimada et al. [58]	MCAO	Wistar rats, male (7 weeks)	Treadmill	Early (4th postinjury day)	28 days, 30 min/day. LowEX: 2 m/min for 5 min, 5 m/min for the next 5 min, and 8 m/min for 20 min. HighEX: 8 m/min for 5 min, 11 m/min for 5 min, and 22 m/min for 20 min	195 m (lowEX); 535 m (highEX) (graded protocols)	MCAO + lowEX spent more time exploring the novel object/newly placed object in object recognition/object location tasks than MCAO + SED. MCAO + highEX explored less than MCAO + lowEX. Both EX groups showed longer latency time in a passive avoidance test compared to MCAO + SED. No group differences in locomotor activity in an open field test	EX groups ↓ lesion size. EX groups ↑ number of neurons in the dentate gyrus compared with non-EX groups – higher in the ipsilateral dentate gyrus in MCAO + lowEX than MCAO + highEX. Ipsilateral dentate gyrus MAP-2 levels ↑ in MCAO + lowEX compared to MCAO + SED. Ipsilateral hippocampal MAP-2 ↓ in MCAO + highEX compared with MCAO + lowEX and sham group. Contralaterally, MAP-2 ↓ in CA1 and CA3 in MCAO + highEX compared with MCAO + lowEX

Sim et al. [53]	CCAO	Mongolian gerbils, male (11–13 weeks)	Treadmill	Early (2nd postinjury day)	10 days. 30 min/day. Speed: 2 m/min for the first 5 min, 5 m/min for the next 5 min, and 8 m/min 20 min	195 m (graded protocol)	CCAO + EX showed longer latency times in a step-down avoidance task than CCAO + SED	TUNEL-positive and Caspase-3 positive cells ↓ in CCAO + EX compared to CCAO + SED. Cell proliferation ↑ in CCAO + SED and sham + EX

Sim et al. [54]	CCAO	Mongolian gerbils, male (11–13 weeks)	Treadmill	Early (1st postinjury day)	4 weeks, 30 min/day. Speed: 2 m/min for the first 5 min, 5 m/min for the next 5 min, and 8 m/min 20 min	195 m (graded protocol)	CCAO + EX showed longer latencies in a step-down avoidance task than CCAO + SED	TUNEL-positive and Caspase-3 positive cells ↓ in CCAO + EX compared to CCAO + SED

Song et al. [51]	PT	Sprague-Dawley rats, male (10 weeks)	Swimming	Early (1 day postinjury)	4 weeks, 5 days/week, 20 min/day	N/A	No significant differences between any treatment groups and lesioned controls on a spatial acquisition task	Hippocampal SOD-levels ↑ and MDA-levels ↓ in all treatment groups compared to lesioned controls. Number of cells in CA3 ↑ in the treatment groups compared to controls

Winocur et al. [44]	IRR	Long Evans rats, male (5 months)	Running wheel	Late (25th postinjury day)	25 days total, 24-hour daily access	IRR + EX: 8.4 km; sham + EX: 3.6 km	No group differences in visual discrimination task acquisition. IRR + EX in high-interference group performed significantly better in retention task than IRR + SED in low-interference group. Sham + EX performed significantly better in retention task than Sham + SED	Exercise ↑ hippocampal DCX and ki67 in all groups

Wong-Goodrich et al. [43]	IRR	C57BL/6 mice, female (8 weeks)	Running wheel	Late (1 month postinjury)	111 days total, 8–12-hour daily access	IRR + EX: 0.56 km; sham + EX: 0.52 km	IRR + EX: longer latency to complete spatial acquisition task on day 1; by day 3 no group differences. IRR + EX also showed improved task retention. No group differences in tail suspension test	No group differences in dentate gyrus size. Hippocampal BrdU- and NeuN-positive cells ↑ in IRR + EX. Levels of TNF-*α*, INF-*γ*, and IL-6 ↑ in IRR groups. Levels of IGF ↑ in IRR + EX compared to IRR + SED. Levels of BDNF and VEGF ↓ in IRR + EX and IRR + SED. Running partially restored VEGF-levels in IRR

Wu et al. [38]	FPI	Sprague-Dawley rats, N/A (200–240 g)	Running wheel	Early (postinjury day 0)	12 days total, daily access: N/A	N/A	FPI + EX + REG and FPI + SED + DHA did significantly better in a spatial acquisition task compared to FPI + SED + REG. FPI + EX + DHA did significantly better than all other FPI groups	Levels of DHA, Acox1, 17*β*-HSD4, Sir2, iPLA2, and p-TrkB ↑ and 4-HHE ↓ in FPI groups subjected to either EX or DHA; this was even more pronounced in FPI + EX + DHA compared to FPI + SED + REG. Levels of STX-3 and BDNF ↑ in FPI + EX + DHA

^*∗*^Early: postinjury days 0–6; late: postinjury days 7 and onwards. ^†^Data from personal communication with corresponding author.

CCAO: common carotid artery occlusion; CCI: controlled cortical impact; CHI: closed head injury; DHA: docosahexaenoic acid diet; earlyEX: early initiated exercise; EX: exercised animals; FPI: fluid percussion injury; highEX: high intensity exercise; IRR: irradiation; lateEX: late initiated exercise; lowEX: low intensity exercise; MCAO: middle cerebral artery occlusion; ModEX: moderate intensity exercise; N/A: information not available; NTI: neurotoxic injury; PT: photothrombosis; REG: regular diet; RW: running wheel exercise; SED: sedentary (nonexercised) animals; Sham: nonlesioned animals; SWIM: swimming exercise.
